# Rotating curved spacetime signatures from a giant quantum vortex

**DOI:** 10.1038/s41586-024-07176-8

**Published:** 2024-03-20

**Authors:** Patrik Švančara, Pietro Smaniotto, Leonardo Solidoro, James F. MacDonald, Sam Patrick, Ruth Gregory, Carlo F. Barenghi, Silke Weinfurtner

**Affiliations:** 1 School of Mathematical Sciences, University of Nottingham, Nottingham, UK; 2 Nottingham Centre of Gravity, University of Nottingham, Nottingham, UK; 3 School of Physics & Astronomy, University of Nottingham, Nottingham, UK; 4 Department of Physics, King’s College London, University of London, London, UK; 5 Perimeter Institute, Waterloo, Ontario Canada; 6 School of Mathematics, Statistics and Physics, Newcastle University, Newcastle upon Tyne, UK; 7 Centre for the Mathematics and Theoretical Physics of Quantum Non-Equilibrium Systems (CQNE), University of Nottingham, Nottingham, UK

**Keywords:** Quantum fluids and solids, Quantum simulation

## Abstract

Gravity simulators^1^ are laboratory systems in which small excitations such as sound^2^ or surface waves^3,4^ behave as fields propagating on a curved spacetime geometry. The analogy between gravity and fluids requires vanishing viscosity^2–4^, a feature naturally realized in superfluids such as liquid helium or cold atomic clouds^5–8^. Such systems have been successful in verifying key predictions of quantum field theory in curved spacetime^7–11^. In particular, quantum simulations of rotating curved spacetimes indicative of astrophysical black holes require the realization of an extensive vortex flow^12^ in superfluid systems. Here we demonstrate that, despite the inherent instability of multiply quantized vortices^13,14^, a stationary giant quantum vortex can be stabilized in superfluid ^4^He. Its compact core carries thousands of circulation quanta, prevailing over current limitations in other physical systems such as magnons^5^, atomic clouds^6,7^ and polaritons^15,16^. We introduce a minimally invasive way to characterize the vortex flow^17,18^ by exploiting the interaction of micrometre-scale waves on the superfluid interface with the background velocity field. Intricate wave–vortex interactions, including the detection of bound states and distinctive analogue black hole ringdown signatures, have been observed. These results open new avenues to explore quantum-to-classical vortex transitions and use superfluid helium as a finite-temperature quantum field theory simulator for rotating curved spacetimes^19^.

## Abstract (web summary)

By stabilizing a stationary giant quantum vortex in superfluid ^4^He and introducing a minimally invasive way to characterize the vortex flow, intricate wave–vortex interactions are shown to simulate black hole ringdown physics.

## Main

To experimentally realize a curved spacetime such as a black hole requires a specific relative motion between the excitations and the background medium. One-dimensional supersonic flow, the archetypal example of an acoustic black hole, provides a platform for observations of Hawking radiation in both classical^20,21^ and quantum fluids^9,10,22^. More complex phenomena such as Penrose superradiance require rotating geometries realizable in two spatial dimensions, for example, by means of a stationary draining vortex flow^12,23^. Classical fluid flow experiments have demonstrated the power of the gravity simulator programme, realizing superradiant amplification of both coherent^11,24^ and evanescent waves^25^, as well as quasinormal mode oscillations^26^, a process intimately connected to black hole ringdown^27^.

Here we investigate related phenomena in the limit of negligible viscosity in superfluid ^4^He (called He II). Its energy dissipation is dependent on temperature and can be finely adjusted across a wide range. At 1.95 K, at which our experiments take place, its kinematic viscosity is reduced by a factor of 100 compared with water^28^ and the damping is dominated by thermal excitations collectively described by the viscous normal component^28,29^ that constitutes approximately half of the total density of the liquid. Moreover, He II supports the existence of line-like topological defects called quantum vortices. Each vortex carries a single circulation quantum *κ* ≈ 10^−7^ m^2^ s^−1^ and forms an irrotational (zero-curl) flow field in its vicinity^29^. Owing to this discretization, a draining vortex of He II can manifest itself only as a multiply quantized (also known as giant) vortex or as a cluster of single quantum vortices. Such vortex bundles exhibit their own collective dynamics and can even introduce solid-body rotation^30^ at length scales larger than the inter-vortex distance, adding complexity to the study of quantum fluid behaviour. As the realization of curved spacetime scenarios requires an irrotational velocity field^1,31^, it is critical to confine any rotational elements into a central area, that is, the vortex core. However, alike-oriented vortices have a tendency to move apart from each other, which poses a limitation on the extent of the core one can stabilize in an experiment. On the other hand, recent findings show that mutual friction^29^ between quantum vortices and the normal component contributes to the stabilization of dense vortex clusters^32^.

The vortex induces a specific velocity field within the superfluid, which affects the propagation of small waves on its surface. In particular, low-frequency excitations perceive an effective acoustic metric^3,4^

1 $${g}_{ij}\propto \left(\begin{array}{rc}-{c}^{2}+{v}^{2} & -{\bf{v}}\\ -{\bf{v}} & {{\mathbb{1}}}_{2\times 2}\end{array}\right),$$

in which *c* denotes their propagation speed and ${\bf{v}}(r,\theta )={v}_{r}\widehat{{\bf{r}}}+{v}_{\theta }\widehat{{\boldsymbol{\theta }}}$ indicates the velocity field at the interface (we assume that the superfluid and normal velocity fields are equal, in line with other mechanically driven flows of He II (refs. ^33,34^)). Although this description fails in the high-frequency regime owing to dispersion, it is well known that the curved spacetime phenomenology persists for these excitations^24,26,35^. Altogether, the above properties suggest that an extensive draining vortex of He II is a feasible candidate for simulations of a quantum field theory in curved spacetime.

**Fig. 1:**
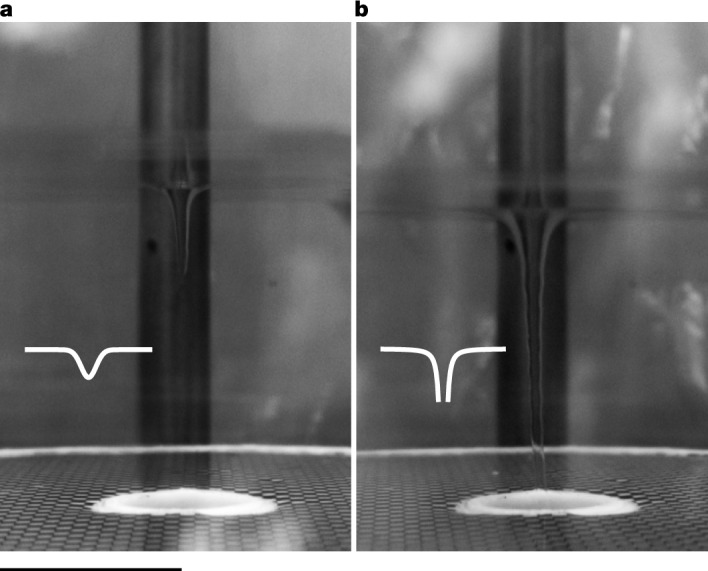
Side views of two distinct configurations of the giant quantum vortex. **a**, At low propeller frequencies (here 1 Hz), the interface exhibits a discernible depression, and the vortex core beneath takes the form of a compact, polarized cluster of singly quantized vortices (called solid core). **b**, With the escalation of frequency (here to 2 Hz), a fully formed hollow core emerges, behaving like a multiply quantized object. Dark vertical stripes in the background provide contrast to the imaged interface. A simplified sketch of this interface (white lines) helps to identify these regimes in later figures. Scale bar, 10 mm.

We realized this flow in cylindrical geometry that is built on the concept of a stationary suction vortex^36^ (see Methods for a detailed description). The central component of our set-up is a spinning propeller, which is responsible for establishing a continuous circulating loop of He II, feeding a draining vortex that forms in the optically accessible experimental zone. At small propeller speeds, we observe a depression on the superfluid interface (Fig. 1a), but as the speed increases, this depression deepens and eventually transforms into a hollow vortex core extending from the free surface to the bottom drain (Fig. 1b). The parabolic shape of the free surface in the former regime is consistent with solid-body rotation, which corresponds to a compact, polarized cluster of singly quantized vortices (called solid core) that forms under the finite depression. The hollow core can instead absorb individual circulation quanta and behave like a multiply quantized object^37^. To minimize the rotational flow injected by the spinning propeller into the experimental zone, we devised a unique recirculation strategy based on a purpose-built flow conditioner (see Methods) that promotes formation of a centrally confined vortex cluster instead of a sparse vortex lattice. However, the exact dynamics of individual quantum vortices, as well as their spatial distribution in the experiment, calls for future investigations. State-of-the-art numerical models^38^ account for the motion of vortex lines coupled to the superfluid and normal velocity fields, but fail to dynamically model the interface, which is a pivotal element in our system. Previous experimental efforts^39–41^ confirmed that a draining vortex in He II carries macroscopic circulation but lacked spatial resolution required to investigate central confinement of rotational components. In this regard, cryogenic flow visualization^42^ provides sufficient resolution. However, this method requires introducing small solid particles into the superfluid, which accumulate along the vortex lines and considerably affect their dynamics^43^.

The above limitations compelled us to propose an alternative, minimally invasive method to examine the vortex flow and extract macroscopic flow parameters that exploit the relative motion occurring between interface waves and the underlying velocity field. The corresponding dispersion relation for angular frequencies *ω* and wave vectors **k** reads^35^

2 $${(\omega -{\bf{v}}\cdot {\bf{k}})}^{2}=F(\parallel {\bf{k}}\parallel ),$$

in which *F* denotes the dispersion function. By solving equation (2), we find (see Methods) that the spectrum of interface modes gets frequency shifted and the velocity field can be inferred from these shifts^44^. Therefore, we redirect our attention towards precise detection of small waves propagating on the superfluid interface.

**Fig. 2:**
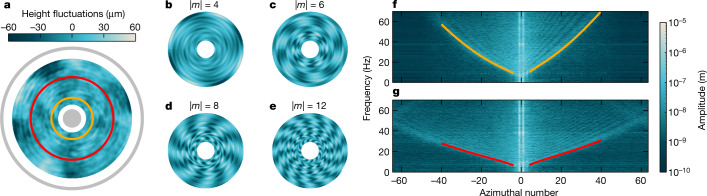
Superfluid interface reconstruction and wave analysis. **a**, Snapshot of the free helium surface depicts height fluctuations representing micrometre waves excited on the superfluid interface. Grey areas mark the positions of the central drain (radius 5 mm) and the outer glass wall (radius 37.3 mm). **b**–**e**, Examples of different azimuthal modes |*m*| (*m* counting the number of wave crests or troughs around a circular path) extracted from panel **a** by a discrete Fourier transform. Wave amplitudes are rescaled for better visibility. **f**,**g**, Two-dimensional wave spectra obtained by transforming angle and time coordinates, for radii of 11.2 mm (panel **f**) and 22.1 mm (panel **g**). These radii are marked in panel **a** by coloured circles. Absence of excitations in low-frequency bands (below the coloured lines) can be understood through the solution of equation (2). The corresponding theoretical predictions of the minimum frequency permissible for propagation for the given radii can be matched with experimental observations (yellow and red lines).

We identified that the adapted Fourier transform profilometry^17,18^ is well suited to our needs, as it is capable of resolving a fluid interface with sufficient and simultaneous resolution in both space and time. This powerful technique consists of imaging the disturbed interface against a periodic backdrop pattern. This way, we resolve height fluctuations of said interface (Fig. 2a) with sensitivity up to approximately one micrometre. Owing to symmetries of the flow, the waves exhibit two conserved quantities: frequency *f* and azimuthal number *m*. The latter parameter counts the number of wave crests around a circular path, with positive or negative values of *m* corresponding to wave patterns co-rotating or counter-rotating with the central vortex.

These spatial patterns (or modes) can be retrieved from the height-fluctuation field by a discrete Fourier transform. For example, by transforming with respect to the angle *θ*, we can single out individual azimuthal modes (Fig. 2b–e). To study wave dynamics in time, we must also transform the temporal coordinate and inspect the resulting two-dimensional spectra, showcased in Fig. 2f,g for two distinct radii. Notable high-amplitude signals in the *m* = ±1 bands are exclusively a consequence of how mechanical vibrations of the set-up imprint themselves on our detection method. Of physical interest are modes with higher azimuthal numbers. These excitations, observed in both solid-core and hollow-core regimes, represent micrometre waves excited on the interface. In the steady state, the waves dissipate their energy, in part by viscous damping and in part by scattering into the draining core of the vortex^45^. Although this is balanced by the stochastic drive originating from the fluid flow and/or aforementioned mechanical vibrations, we notice that only a certain region of the spectral space (*m*, *f*) is populated with excitations, a feature that varies when examining smaller (Fig. 2f) and larger (Fig. 2g) radii. We observe that only some high-frequency (equivalent to high-energy) waves have the capability to propagate on the interface. Through the solution of equation (2), we can pinpoint the minimum frequency, *f*_min_, permissible for propagation for the given radius, azimuthal number and background velocity (see Methods) and, in line with the methodology introduced above, we exploit this particular frequency to extract the underlying velocity field, as we now describe. We search the parameter space produced by two velocity components (*v*_*r*_, *v*_*θ*_) and determine values that produce the best match between *f*_min_ and the lowest excited frequency in the experimental data across several azimuthal modes (coloured lines in Fig. 2f,g). By carrying out this procedure for every examined radius, we can reconstruct the velocity distribution in the draining vortex flow.

**Fig. 3:**
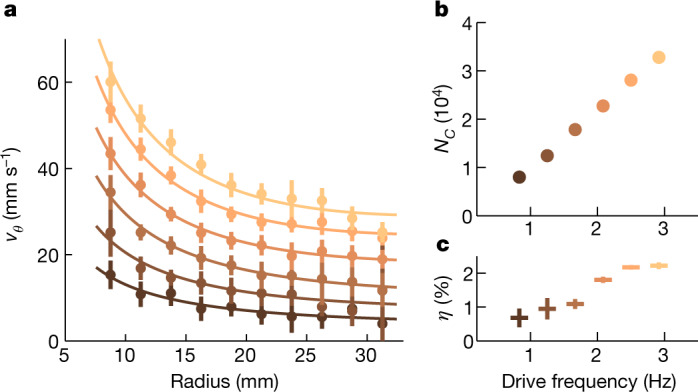
Reconstructed velocity distribution and flow parameters. **a**, Coloured points denote the radial dependence of the azimuthal velocity *v*_*θ*_ for six vortex configurations distinguished by the drive (propeller) frequency. Each point is obtained by averaging over a 2.5-mm radial interval. Radial velocity component is approximately zero across all instances. Best fits of *v*_*θ*_(*r*) (coloured lines) yield the circulation *C* of the central vortex and the angular frequency *Ω* of the extra solid-body rotation. **b**, Number of circulation quanta confined in the vortex core, *N*_*C*_ = 2π*C*/*κ*, corresponds to the most extensive vortex structures ever observed in quantum fluids. **c**, The ratio *η* between *Ω* and the angular frequency of the drive is less than 2.5% in all cases, suggesting that the velocity field in our system is dominated by the irrotational vortex flow. Vertical error bars in panels **a** and **c** denote one standard deviation intervals. Standard deviation intervals of data points in panel **b**, comparable with the symbol size, are not shown.

We conducted these reconstructions across several vortex configurations distinguished by the drive (propeller) frequency. For all instances, *v*_*r*_ approximates zero within the limits of our resolution. Although seemingly paradoxical, this outcome results from a complex boundary-layer interaction and is in agreement with earlier findings in classical fluids^46^. Therefore, interface waves engage with an almost entirely circulating flow characterized by a specific radial dependence of *v*_*θ*_ (coloured points in Fig. 3a). Overall, the results are consistent with

3 $${v}_{\theta }(r)=\varOmega r+\frac{C}{r},$$

indicated in Fig. 3a by coloured lines. The first term represents solid-body rotation with angular frequency *Ω*, which leaks into the experimental area through the flow conditioner as described above. The second term corresponds to an irrotational flow around a central vortex with circulation *C*. The related number of circulation quanta confined in its core, *N*_*C*_ = 2π*C*/*κ*, is shown in Fig. 3b as a function of the drive frequency. Across all instances, the core consists of the order of 10^4^ quanta, a record-breaking value in the realm of quantum fluids. In the solid-core regime, *N*_*C*_ can be identified with the number of individual quantum vortices concentrated in the core. However, in the context of a hollow core, *N*_*C*_ represents its topological charge. Achieving circulation values separated from the elementary quantum *κ* by four orders of magnitude allows the quantization of circulation to be disregarded, leaving the vortex effectively classical. This unprecedented realization of a giant quantum vortex flow represents a distinctive instance of a quantum-to-classical flow transition in He II (ref. ^47^).

The importance of the aforementioned outcomes can be underlined by noting that *n*-quantized vortices are dynamically unstable^13,14^. They spontaneously decay into a cluster of *n* vortices^48^ as a result of the excitation of a negative energy mode in the multiply quantized vortex core^11,48^. Nevertheless, dynamical stabilization of giant vortices can be achieved by suitably manipulating the superfluid. Namely, introducing a draining flow and reducing the fluid density at the centre has proven effective in polariton condensates, for vortices with *n* ≲ 100 (refs. ^15,16^). These results agree with our experiment, in which the reduced density translates into the existence of a hollow core and the draining flow resides in the bulk of the draining vortex.

It is worth noting that larger circulation values around a draining vortex in He II are documented in the literature^41^. However, therein, the contributions of the vortex core and the solid-body rotation are not distinguished. The second effect may dominate in the reported circulations, as the number of quantum vortices responsible for rotation^30^ scales with the corresponding angular frequency *Ω*. Rotation in our experimental zone is notably suppressed. The sparse presence of quantum vortices partially justifies our assumption that normal and superfluid components behave as a single fluid. More importantly, the ratio *η* between *Ω* and the angular frequency of the drive does not exceed 2.5% (Fig. 3c), and the velocity field in our system is dominated by the irrotational vortex flow. The core of this vortex must be smaller than 7.6 mm, the smallest investigated radius, because the velocity profiles (Fig. 3a) show no indication of a turning point at small radii.

**Fig. 4:**
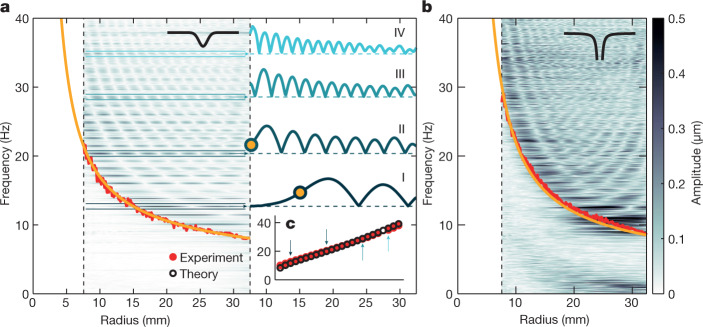
Bound states in co-rotating waves. Fourier amplitudes of interface waves corresponding to *m* = 8 mode show a characteristic pattern in the radial direction that can be identified with bound states, that is, standing waves between the outer boundary (glass wall) at 37.3 mm and the effective potential barrier (red lines). A simplified but accurate model of the potential (yellow lines) is extended beyond the experimentally accessible range (dashed black lines). **a**, Solid-core regime. Rescaled amplitudes of four bound states labelled I–IV (blue lines) are shown as a function of radius. Crossing points with the potential barrier are marked by yellow points. **b**, Hollow-core regime. **c**, Comparison of bound-state frequencies retrieved from panel **a** (red points) and their theoretical predictions (black circles). Frequencies of states I–IV are highlighted by blue arrows.

We can, nonetheless, venture beyond the experimental range by exploring wave dynamics in the radial direction. We restrict our discussion to a particular mode |*m*| = 8 (Fig. 2d) as a representative of the outlined behaviour. We start by analysing co-rotating (*m* = 8) modes, shown in Fig. 4a,b for the solid-core and hollow-core structures. In both cases, *f*_min_ (red line) denotes an effective potential barrier, preventing waves from reaching the vortex core. Existence of this barrier, together with an outer, solid boundary at 37.3 mm, gives rise to bound states (standing waves), appearing as distinct, striped patterns extending up to 40 Hz. These patterns represent the first direct measurement of resonant surface modes around a macroscopic vortex flow in He II.

To perform an in-depth examination of selected states (denoted as I–IV), we plot the absolute value of their amplitudes in Fig. 4a. The frequency of state I meets *f*_min_ in a crossing point (yellow point) located within the field of view. At large radii, this wave harmonically propagates. However, as it penetrates the barrier, its amplitude exponentially decays in exact analogy with a simple quantum-mechanical model of a particle trapped in a potential well. For higher frequencies, the crossing point moves towards smaller radii (state II), eventually reaching the limit of our detection range. For the highest-frequency states (III and IV), the crossing point is well outside the detection range and we only observe the harmonic part of the signal. Nonetheless, the mere existence and predictability of these states lets us extend the effective potential barrier beyond the observable range.

Specifically, we consider a model of a purely circulating vortex, whose velocity field reads (*v*_*r*_, *v*_*θ*_) = (0, *C*/*r*), and extend the experimentally determined potential barrier (red lines in Fig. 4a,b) towards smaller radii (yellow lines). In practice, this model must break down near the vortex core, at which point the spatial distribution of individual quantum vortices becomes relevant. Nonetheless, the frequencies of individual bound states are in excellent agreement with theoretical predictions (see Methods) based on the extended potential barrier (Fig. 4c). This outcome validates the simplified model and allows us to constrain the radius of the core region to approximately 4 and 6 mm, respectively for the solid-core and hollow-core regimes. Confinement of the rotating core beyond the experimental range gains importance when considering the draining vortex flow as a gravity simulator, for example, when searching for initial indications of black hole ringdown.

**Fig. 5:**
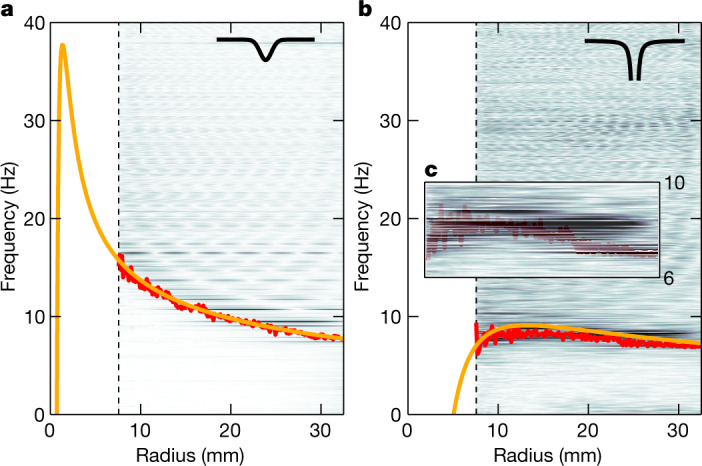
Bound states and ringdown modes in counter-rotating waves. Fourier amplitudes of interface waves (same colour scale as in Fig. 4) corresponding to *m* = −8 mode interact with the effective potential barrier (red lines). Its simplified model (yellow lines) is extended beyond the accessible range (dashed black lines). **a**, In the solid-core regime, the potential allows existence of bound states, visible up to approximately 30 Hz. **b**, In the hollow-core regime, no bound states can be retrieved. Instead, we observe dominant excitations lingering near the shallow maximum of the potential (approximately at 8.25 Hz), suggesting the excitation of black hole ringdown modes. **c**, Inset highlights ringdown mode candidates from panel **b**, with the effective potential barrier shown as a faint red line.

For this purpose, we focus on counter-rotating (*m* = −8) modes, depicted in Fig. 5a,b with the effective potential barriers (red lines) and their extensions (yellow lines). The shape of the barrier in the solid-core regime (Fig. 5a) allows the existence of bound states up to approximately 30 Hz. However, this is not the case in the hollow-core regime (Fig. 5b), despite the corresponding circulations only differing within one order of magnitude. Bound states are not formed at all because the effective potential shows a shallow maximum before decreasing towards zero. Dominant excitations in this spectrum, highlighted in Fig. 5c, are modes lingering near this maximum. These excitations, previously identified as ringdown modes of an analogue black hole^26^, represent the very first hints of this process taking place in a quantum fluid. The radius at which the effective potential crosses the zero-frequency level is related to the analogue ergoregion^35^, a key feature in the occurrence of black hole superradiance. To directly observe this region in our set-up, further increasing the azimuthal velocity and/or examining the system closer to the vortex core is required.

Our research positions quantum liquids, particularly He II, as promising contenders for finite-temperature, non-equilibrium quantum field theory simulations, marking a transformative shift from already established simulators in curved spacetimes^7–10^. The liquid nature of He II arises from an effective, strongly interacting field that complements its weakly interacting counterpart found in, for example, cold atomic clouds. A distinctive advantage presented by He II lies in its flexibility, allowing it to be operated at a fixed temperature, starting just below the superfluid transition, at which He II shows pronounced dissipation. This regime in particular holds immense potential, such as for the mapping to generic holographic theories^49^. At temperatures below 1 K, the normal component is expected to be an aggregate of individual thermal excitations. This tunability provides the opportunity to investigate a broad range of finite-temperature quantum field theories.

Owing to the capacity of He II to accommodate macroscopic systems, we achieved the creation of extensive vortex flows in a quantum fluid. Notably, the size of the hollow vortex core scales with its winding number and, consequently, system-size constraints may restrict the maximum circulation achievable when implemented in cold-atom or polariton systems alike. Key processes in rotating curved spacetimes, such as superradiance and black hole ringing, can be explored in our current system with minor adjustments to the propeller speed, container geometry or by dynamically varying flow parameters. Our set-up also provides a clear opportunity to investigate rotating curved spacetimes with tunable and genuinely quantized angular momentum, setting it apart from classical liquids. Furthermore, applying these techniques to explicitly time-dependent scenarios allows for the exploration of fundamental non-equilibrium field theory processes. This may involve controlled modulations of first or second sound in the bulk of the quantum liquid, providing a platform for conducting wave-turbulence simulations across various length and temperature scales. This represents a noteworthy advancement beyond the current scope of cold-atom studies^50^.

## Methods

### Cryogenic drive

**Extended Data Fig. 1:**
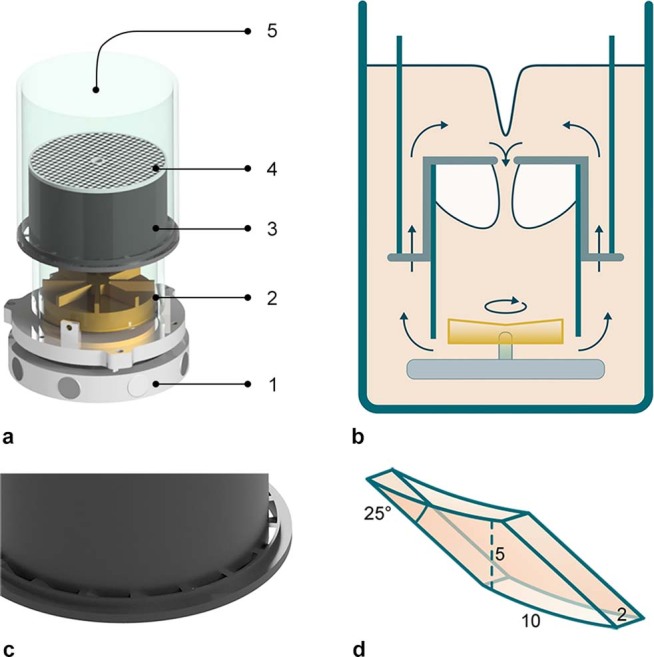
Cryogenic realization of a stationary giant quantum vortex. **a**, A disc (1) is magnetically coupled to a room-temperature drive to achieve rotation frequencies of up to 3 Hz. The disc drives a propeller (2) functioning as a centrifugal pump, establishing a continuous circulation loop of He II through a purpose-built flow conditioner (3) decorated with a periodic pattern (4). A draining vortex forms in the centre of the designated experimental zone (5). **b**, Schematic illustrating the outline of our recirculation technique. **c**, Detail on the edge of the flow conditioner equipped with 18 slanted openings. **d**, Schematic representation of one such opening, with dimensions given in millimetres.

The experimental set-up (Extended Data Fig. 1a) relies on the use of a spinning propeller that acts as a centrifugal pump, establishing a steady recirculation loop of He II between the experimental zone and the area underneath (Extended Data Fig. 1b). The draining vortex forms above a circular aperture, 10 mm in diameter and located at the top of a purpose-built flow conditioner (Extended Data Fig. 1c), whose geometry was optimized through experimentation. Namely, the flow conditioner features 18 slanted openings (Extended Data Fig. 1d) regularly placed along its outer edge, which are used to reinject He II into the experimental zone. To suppress the amount of undesired solid-body rotation that leaks into the experimental zone from the vicinity of the propeller, we adopt the strategy of driving the propeller in the direction opposite to the orientation of these openings. We eventually find that the direction of the draining vortex that forms in the experimental zone coincides with the direction of these openings.

To establish a stable recirculation of He II, it is crucial to rely on a constant drive of the propeller. For this purpose, a robust connection between the propeller submerged in He II and its room-temperature drive is achieved by means of magnetic coupling. In particular, a magnetic ring rotates outside the cryostat driven by a DC motor. Inside the cryostat, a solid disc decorated with permanent magnets (see again Extended Data Fig. 1a, 1) ensures that motion is transferred to the propeller (Extended Data Fig. 1a, 2). This way, we achieve a smooth operation of the drive, with steadfast connection up to approximately 3 Hz, ensuring continuous and reliable power transmission to the low-temperature environment. The need for magnetic coupling led us to enclose the set-up in a custom cryogenic system made of glass. Its fully transparent design provides excellent optical access, while maintaining a constant temperature of the helium bath within the range down to 1.55 K and with stability better than 1 mK.

### Interface wave dynamics

We consider a potential flow **v** = ∇Φ and a free surface located at *z* = *h*_0_. Perturbations of the velocity potential and free surface are denoted *δ*Φ and *δh*, respectively. The equations of motion for surface waves are obtained by linearizing the Bernoulli equation and integrating Laplace’s equation from the hard boundary at *z* = 0 to the free surface,

4 $$\begin{array}{l}({\partial }_{t}+{\bf{v}}\cdot \nabla )\delta \Phi +g\delta h-\frac{\sigma }{\rho }{\nabla }^{2}\delta h=0,\\ ({\partial }_{t}+\nabla \cdot {\bf{v}})\delta h+i\nabla \cdot \tanh (-i{h}_{0}\nabla )\delta \Phi =0,\end{array}$$

in which *g* is the gravitational acceleration, *σ* = 3.06 × 10^−4^ N m^−1^ is the surface tension and *ρ* = 145.5 kg m^−3^ is the density of the fluid (numerical values are given for He II at 1.95 K (ref. ^28^)). These equations can be derived from the following action,

5 $$\begin{array}{l}S[\delta \Phi ,\delta h]\,=\,\int \,{\rm{d}}t\,{{\rm{d}}}^{2}{\bf{x}}\left[-\delta h({\partial }_{t}+{\bf{v}}\cdot \nabla )\delta \Phi -\frac{1}{2}g\delta {h}^{2}\right.\\ \,\,\,\,\,\left.-\frac{\sigma }{2\rho }{| \nabla \delta h| }^{2}-\frac{1}{2}\delta \Phi {\mathcal{D}}(-i\nabla )\delta \Phi \right],\end{array}$$

in which ${\mathcal{D}}({\bf{k}})=k\tanh ({h}_{0}k)$ and *k* = ||**k**||. This constitutes the effective field theory of our system once the bulk degrees of freedom have been integrated out. For *h*_0_*k* ≪ 1, the effect of the inhomogeneous fluid flow is completely encapsulated by the effective metric given in equation (1) (ref. ^1^). Wavelengths in our system satisfy the opposite limit, *h*_0_*k* ≫ 1. In this regime, solution of equation (4) can be achieved using the Wentzel–Kramers–Brillouin (WKB) approximation, for which the local wavelength is assumed to be much shorter than the scale over which the background flow varies.

Within the WKB approximation, the solution of equation (4) is modelled as a plane wave with an amplitude and wave vector that vary smoothly as we move through the system. Assuming that **v** only depends on the radial coordinate and recalling that the angular frequency *ω* and the azimuthal number *m* are conserved quantities within our system, we can write

6 $$\delta \Phi \propto \exp (i\int p(r){\rm{d}}r+im\theta -i\omega t),$$

in which *p*(*r*) is the radial part of the wave vector ${\bf{k}}=p(r)\widehat{{\bf{r}}}+(m/r)\widehat{{\boldsymbol{\theta }}}$. The amplitude is similarly only a function of *r*. At leading order, the WKB method gives the local dispersion relation, see equation (2), with the dispersion function,

7 $$F(k)=\left(gk+\frac{\sigma }{\rho }{k}^{3}\right)\tanh ({h}_{0}k),$$

which is only a function of the modulus of the wave vector *k*.

As discussed in the main text, the background flow can be accurately modelled as ${\bf{v}}=(C/r)\widehat{{\boldsymbol{\theta }}}$. By further assuming that *h*_0_ is constant in the window of analysis, the solutions of equation (2) define two branches of the dispersion relation,

8 $${\omega }_{D}^{\pm }=\frac{mC}{{r}^{2}}\pm \sqrt{F(k)}.$$

We are interested in the behaviour of equation (8) as a function of the azimuthal number *m*. For *m* ≠ 0, we can distinguish two different contributions: one explicitly appears as *mC*/*r*^2^, whereas the second is hidden in the modulus of **k**, which is proportional to *m*^2^/*r*^2^. The effect of the latter is to widen the gap between ${\omega }_{D}^{+}$ and ${\omega }_{D}^{-}$ when approaching the vortex core, that is, when decreasing *r*. The *mC*/*r*^2^ term shifts both branches up for *m* > 0 or down for *m* < 0. For large enough ||**v**||, it is possible for ${\omega }_{D}^{-}$ to cross into the upper half plane (*ω* > 0) for *m* > 0 or for ${\omega }_{D}^{+}$ to cross into the lower half plane (*ω* < 0) for *m* < 0. However, this does not occur in our window of analysis, which excludes the vortex core, and therefore ${\rm{sgn}}({\omega }_{D}^{\pm })=\pm 1$ for all *r* and *p* considered. From here on, we restrict our attention to positive frequency modes, for which only ${\omega }_{D}^{+}$ is relevant.

For a given angular frequency *ω*, the solutions of the dispersion relation $\omega ={\omega }_{D}^{+}(m,r,p)$ at a particular radius determine the allowed values of the radial wave vector *p*. There are a maximum of two solutions with $p\in {\mathbb{R}}$ for the considered flow profile, which correspond to the (radially) in-going and out-going waves far away from the vortex core. The function ${\omega }_{D}^{+}$ presents a minimum frequency *ω*_min_ below which there are no intersections between a line of constant *ω* with ${\omega }_{D}^{+}$ at any real *p*. Therefore, *ω*_min_ sets the minimum frequency for a wave to be able to propagate in the system at that particular radius. We can find this frequency by solving ${\partial }_{p}{\omega }_{D}^{+}=0$, which gives *p* = 0. By inserting the solution into the dispersion relation, we obtain ${\omega }_{\min }(m,r)={\omega }_{D}^{+}(m,r,p=0)$, which (after dividing by 2π) gives the yellow lines shown in Figs. 4a,b and 5a,b.

For *ω* < *ω*_min_, $p\in {\mathbb{C}}$ and the solutions become evanescent. In line with the main text, *ω*_min_ doubles as an effective potential barrier, trapping any propagating solutions in the radial direction between the crossing point *r*_tp_ (defined by *ω* = *ω*_min_(*r*_tp_)) and the outer boundary at *r*_B_. The crossing point can be also understood as the turning point of the dispersion relation, that is, the location at which an in-going wave comes to rest (as seen from the vanishing of the radial part of the group velocity, ${\partial }_{p}{\omega }_{D}^{+}({r}_{{\rm{tp}}})=0$) and gets reflected. When this happens, the two roots *p*^±^ of the positive form of equation (8) become degenerate and there is a divergence in the WKB amplitude, causing this approximation to break down. However, there is a known procedure^35^ to circumvent this pathological behaviour, as we now describe.

The WKB solution in the vicinity of an arbitrary point *r*_*j*_ may be written as

9 $$\delta \Phi \propto {\alpha }_{j}^{+}\,\exp \left(i{\int }_{{r}_{j}}^{r}p({r}^{{\prime} }){\rm{d}}{r}^{{\prime} }\right)+{\alpha }_{j}^{-}\,\exp \left(-i{\int }_{{r}_{j}}^{r}p({r}^{{\prime} }){\rm{d}}{r}^{{\prime} }\right),$$

in which ${\alpha }_{j}^{\pm }$ are constants and we have omitted the radially dependent prefactor shared by both terms, as well as an overall factor of exp(*imθ* − *iωt*), which are irrelevant for our purposes. At *r*_B_, we have the requirement that no fluid penetrates the outer boundary,

10 $${\partial }_{r}\delta \Phi ({r}_{{\rm{B}}})=0,$$

which is solved by setting ${\alpha }_{{\rm{B}}}^{+}={\alpha }_{{\rm{B}}}^{-}$. In the vicinity of *r*_tp_, it is possible to solve the wave equation (4) directly to find a relation between the WKB amplitudes. However, if the evanescent mode decays as it moves away from the turning point (manifestly apparent for state I in Fig. 4a), the relevant relation simply reads ${\alpha }_{{\rm{tp}}}^{-}=i{\alpha }_{{\rm{tp}}}^{+}$. Relating the amplitudes on either side of *r*_tp_, one obtains the resonance condition,

11 $${\int }_{{r}_{{\rm{tp}}}}^{{r}_{{\rm{B}}}}p({r}^{{\prime} }){\rm{d}}{r}^{{\prime} }=\pi \left(n+\frac{1}{4}\right),$$

in which *n* = 0, 1, 2… indexes possible bound states that fit inside the cavity delineated by *r*_tp_ and *r*_B_. We solve this condition to find the various *ω*_*n*_ and show the corresponding frequencies as open black circles in Fig. 4c.

## Online content

Any methods, additional references, Nature Portfolio reporting summaries, source data, extended data, supplementary information, acknowledgements, peer review information; details of author contributions and competing interests; and statements of data and code availability are available at 10.1038/s41586-024-07176-8.

## Data Availability

The datasets generated and analysed during the current study are available from the corresponding author.

## Code availability

The conclusions of this study do not depend on code or algorithms beyond standard numerical evaluations.
